# Genetic architecture of human thinness compared to severe obesity

**DOI:** 10.1371/journal.pgen.1007603

**Published:** 2019-01-24

**Authors:** Fernando Riveros-McKay, Vanisha Mistry, Rebecca Bounds, Audrey Hendricks, Julia M. Keogh, Hannah Thomas, Elana Henning, Laura J. Corbin, Understanding Society Scientific Group, Stephen O’Rahilly, Eleftheria Zeggini, Eleanor Wheeler, Inês Barroso, I. Sadaf Farooqi

**Affiliations:** 1 Wellcome Sanger Institute, Cambridge, United Kingdom; 2 University of Cambridge Metabolic Research Laboratories and NIHR Cambridge Biomedical Research Centre, Wellcome Trust-MRC Institute of Metabolic Science, Addenbrooke’s Hospital, Cambridge, United Kingdom; 3 Department of Mathematical and Statistical Sciences, University of Colorado-Denver, Denver, Colorado, United States of America; 4 MRC Integrative Epidemiology Unit at University of Bristol, Bristol, United Kingdom; 5 Population Health Sciences, Bristol Medical School, University of Bristol, Bristol, United Kingdom; Washington University in Saint Louis School of Medicine, UNITED STATES

## Abstract

The variation in weight within a shared environment is largely attributable to genetic factors. Whilst many genes/loci confer susceptibility to obesity, little is known about the genetic architecture of healthy thinness. Here, we characterise the heritability of thinness which we found was comparable to that of severe obesity (h^2^ = 28.07 vs 32.33% respectively), although with incomplete genetic overlap (r = -0.49, 95% CI [-0.17, -0.82], *p* = 0.003). In a genome-wide association analysis of thinness (n = 1,471) vs severe obesity (n = 1,456), we identified 10 loci previously associated with obesity, and demonstrate enrichment for established BMI-associated loci (*p*_*binomial*_ = 3.05x10^-5^). Simulation analyses showed that different association results between the extremes were likely in agreement with additive effects across the BMI distribution, suggesting different effects on thinness and obesity could be due to their different degrees of extremeness. In further analyses, we detected a novel obesity and BMI-associated locus at *PKHD1* (rs2784243, obese vs. thin *p* = 5.99x10^-6^, obese vs. controls *p* = 2.13x10^-6^ *p*_*BMI*_ = 2.3x10^-13^), associations at loci recently discovered with much larger sample sizes (e.g. *FAM150B* and *PRDM6-CEP120*), and novel variants driving associations at previously established signals (e.g. rs205262 at the *SNRPC*/*C6orf106* locus and rs112446794 at the *PRDM6-CEP120* locus). Our ability to replicate loci found with much larger sample sizes demonstrates the value of clinical extremes and suggest that characterisation of the genetics of thinness may provide a more nuanced understanding of the genetic architecture of body weight regulation and may inform the identification of potential anti-obesity targets.

## Author summary

Obesity-associated disorders are amongst the leading causes of morbidity and mortality worldwide. Most genome-wide association studies (GWAS) have focused on body mass index (BMI = weight in Kg divided by height squared (m^2^)) and obesity, but to date no genetic association study testing thin and healthy individuals has been performed. In this study, we recruited a first of its kind cohort of 1,471 clinically ascertained thin and healthy individuals and contrasted the genetic architecture of the trait with that of severe early onset obesity. We show that thinness, like obesity, is a heritable trait with a polygenic component. In a GWAS of persistent healthy thinness vs. severe obesity with a total sample size of 2,927, we are able to find evidence of association in loci that have only been recently discovered using large cohorts with >40,000 individuals. We also find a novel BMI-associated locus at *PKHD1* in UK Biobank highlighted by our association study. This work illustrates the value and increased power brought upon by using clinically ascertained extremes to study complex traits and provides a valuable resource on which to study resistance to obesity in an increasingly obesogenic environment.

## Introduction

The rising prevalence of obesity is driven by changes in the environment including the consumption of high calorie foods and reduced levels of physical activity [1]. However, within a given environment, there is considerable variation in body weight; some people are particularly susceptible to severe obesity, whilst others remain thin [2,3]. Family, twin and adoption studies have consistently demonstrated that 40–70% of the variation in body weight can be attributed to heritable factors [4]. As a result, many studies have focused on the genetic basis of body mass index (BMI) and/or obesity. To date >250 common and low-frequency obesity-susceptibility loci have been identified [5–10]. Additionally, studies of people at one extreme of the distribution (severe obesity) have led to the identification of rare, penetrant genetic variants that affect key molecular and neural pathways involved in human energy homeostasis [11–14]. These findings have provided a rationale for targeting these pathways for therapeutic benefit. In contrast, little is known about the specific genetic characteristics of persistently thin individuals (thinness defined using WHO criteria BMI≤18kg/m^2^). Understanding the mechanisms underlying thinness/resistance to obesity may highlight novel anti-obesity targets for future drug development.

A small number of previous studies have found that thinness appears to be a trait that is at least as stable and heritable as obesity [15–18]. A large study of 7,078 UK children and adolescents, found that the strongest predictor of child/adolescent thinness was parental weight status. The prevalence of thinness was highest (16.2%) when both parents were thin and progressively lower when both parents were normal weight, overweight or obese [19].

One approach to studying thinness is to study individuals from a population-based cohort for a quantitative or continuous trait. For example, it is possible to generate a “case-control” study by taking the extremes of the population distribution for a continuous trait such as BMI, an approach used effectively by Berndt *et al*. 2013 [20] who analysed the top and bottom 5% in cohorts participating in the GIANT Consortium. However, by their very definition, such population-based cohorts often contain a limited number of people at the “extremes” (i.e. severe obesity and thinness) [20]. To date, other GWAS approaches that included thin individuals have either used them exclusively as controls to contrast with extreme obesity [21], or have not ascertained for healthy thinness [22]. Here, we use a different study design, and one that has been used to increase power to detect genetic association, in particular for disorders where there is a large environmental component (e.g. asthma, type 2 diabetes and obesity), enriching our case series with affected individuals that may be more genetically loaded. This selection is usually done by selecting individuals who may have a more extreme form of disease, are younger (less time for environment to impact their disease) and perhaps have family members also affected with the same condition. To complement this approach to the selection of cases, controls are also selected to increase the chances that they do not have the disease or are unlikely to develop the disease later in life [21]. This is normally done by selecting contrasting controls, or “super-controls”. However, the low prevalence of thinness in countries such as the UK and the fact that people who are well but constitutionally thin do not routinely come to medical attention, poses challenges to recruitment of a cohort of healthy thin individuals. We were able to take advantage of the UK National Health Service (NHS) research infrastructure to recruit from primary care (Methods) using body mass index (BMI: weight in kg/height in metres^2^) criteria and personal review of individual case files to identify a cohort of approximately 2000 UK European descent thin adults (Study Into Lean and Thin Subjects, STILTS cohort; mean BMI = 17.6 kg/m^2^) who are well, without medical conditions or eating disorders (Methods). 74% of the STILTS cohort have a family history of persistent thinness throughout life, suggesting we have enriched for genetically driven thinness.

Here, we present a new, and the largest-to-date, GWAS focused on persistent healthy thinness and contrast the genetic architecture of this trait with that of severe early onset obesity ascertained in the clinic. We explored whether the genetic loci influencing thinness are the same as those influencing obesity, i.e., are these two clinically ascertained traits reverse sides of the same “coin”, or whether there are important genetic differences between them. We show that persistent thinness and severe early onset obesity are both heritable traits (h^2^ = 28.07% and h^2^ = 32.33%, respectively) that share a number of associated loci, and both are enriched for established BMI associated loci (binomial *p* = 3.05x10^-5^ and 9.09x10^-13^, respectively). Nonetheless, we also detected important differences, with some loci more strongly associated at the upper clinical end of the BMI distribution (e.g. *FTO*), some at the lower end (e.g. *CADM2*), whilst other loci are equivalently associated with both clinical ends of the BMI spectrum (e.g. *MC4R*). Simulation tests showed that these results did not significantly deviate from additive effects and most likely reflect the different degrees of extremeness present in our clinically ascertained cohorts, where severely obese individuals represent a more significant deviation from the mean than healthy thin individuals do (the same degree of thinness may not be compatible with healthy human life). These data support expansion of genetic studies of persistent thinness as an approach to gain further insights into the biology underlying human energy homeostasis, and as an alternative approach to uncovering potential anti-obesity targets for drug development.

## Results

### Heritability of persistent thinness and severe early onset obesity

To investigate the heritability of healthy thinness and contrast it with that of severe early onset childhood obesity we obtained genotype data for 1,622 persistently thin healthy individuals (STILTS), 1,985 severe childhood onset obesity cases (SCOOP; European ancestry individuals from the GOOS cohort) and 10,433 population-based individuals (UKHLS) used as a common set of controls (Methods, S1 Table). All participants were genotyped on the Illumina Core Exome array, including 551,839 markers. After sample and variant quality control, we retained 1,471 thin individuals, 1,456 obese individuals, 6,460 control individuals in the BMI range 19–30 kg/m^2^ (non-extremes). 477,288 directly genotyped variants were included in the analysis (Methods); 54% common variants (minor allele frequency (MAF) ≥1% amongst controls) and 46% rare variants (MAF<1% amongst controls), of which most were protein-coding (96.8%). We then imputed genotypes to a combined UK10K+1000G reference panel and, using LD score regression, we estimated that a subset of 1,197,969 HapMap3 markers accounted for 32.33% (95% CI 23.75%-40.91%) of the phenotypic variance on the liability scale in severe early onset obesity, and 28.07% (95% CI 13.80%-42.34%) in persistent thinness, suggesting both traits are similarly heritable (Methods). The heritability estimates reported here were used mainly to establish the fact that thinness is a heritable trait; we expect our liability scale estimates to be mostly unbiased given the study design [23]. However, given the low prevalence of the traits presented here, these estimates may represent upper bounds.

### Contribution of known BMI associated loci to thinness and severe early onset obesity

**Fig 1:**
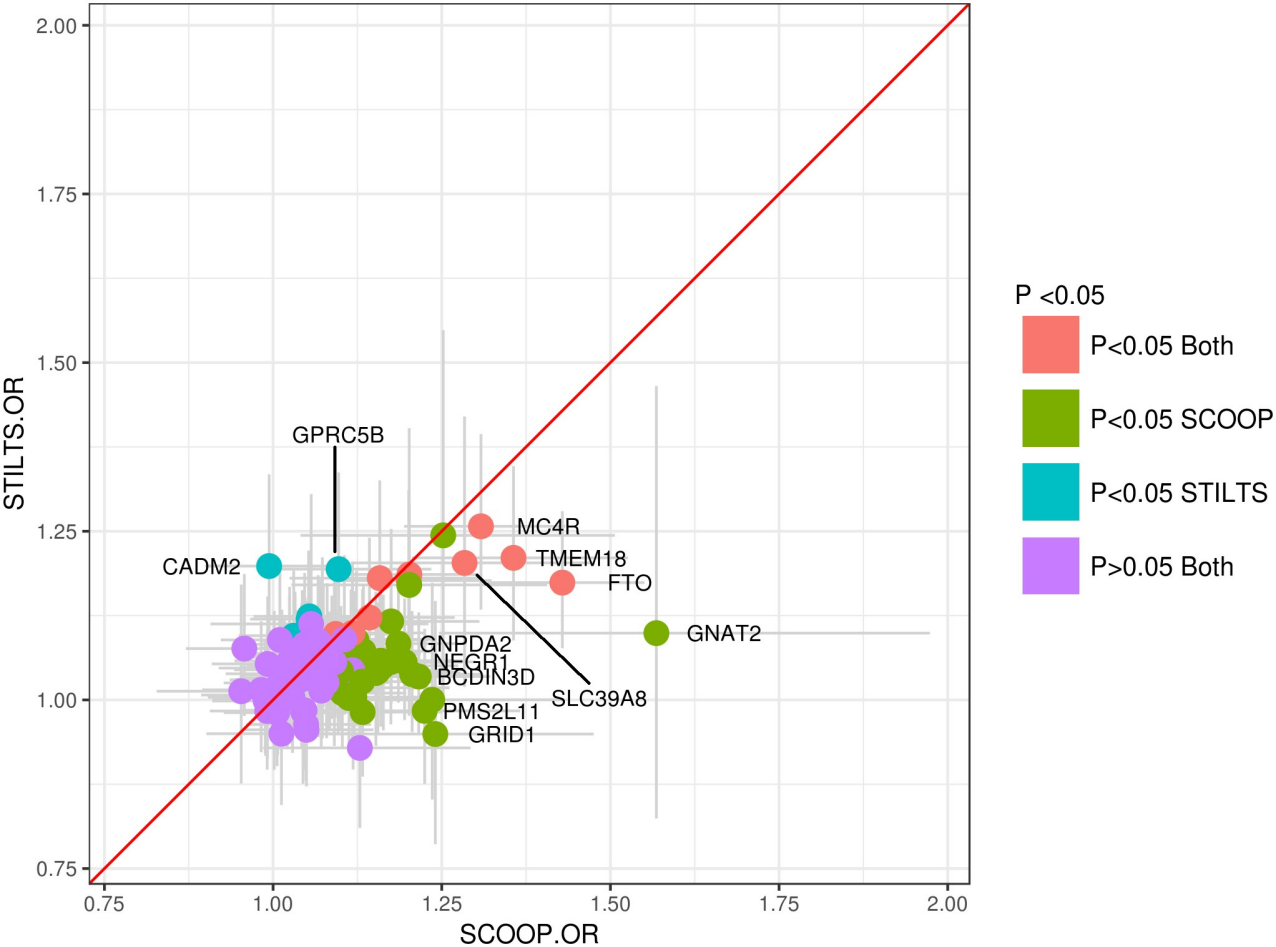
Odds ratio comparison for established BMI associated loci. Odds ratios for SCOOP vs UKHLS (x-axis) and UKHLS vs STILTS (y-axis) comparisons are shown for the 97 known BMI loci from GIANT [24]. Colours of data points represent nominal significance in both analyses (red), only SCOOP vs. UKHLS (green), only STILTS vs UKHLS (blue) or in neither analysis (purple). Error bars represent 95% confidence intervals for the odds ratios for SCOOP vs UKHLS (x-axis) and for UKHLS vs STILTS (y-axis). A subset of data points with larger separation from the red diagonal line (x = y) are labelled.

**Fig 2:**
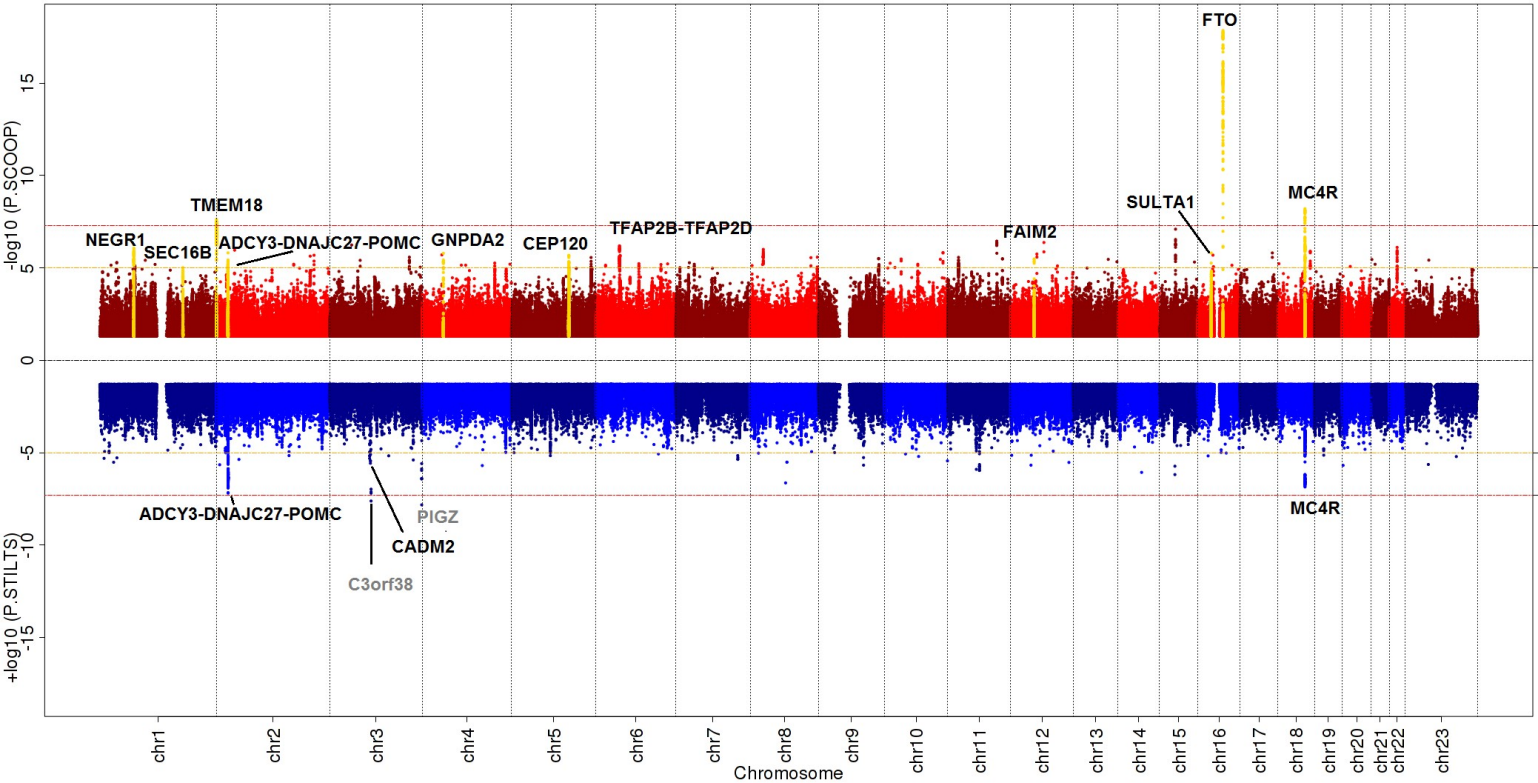
Miami plot of SCOOP vs. UKHLS and STILTS vs. UKHLS. Miami plot produced in EasyStrata [29], Red = SCOOP vs. UKHLS; Blue = STILTS vs. UKHLS. Red lines indicate genome-wide significance threshold at *p* = 5x10^-08^. Orange lines indicate discovery significance threshold at *p* = 1x10^-05^. Black labels highlight known BMI/obesity loci that were taken forward for replication and yellow peaks indicate those that met genome-wide significance after replication. Grey labels highlight novel loci with *p*<5x10^-08^ that did not replicate.

To investigate the role of established common variant European BMI associated loci, we studied the 97 loci from GIANT [24] in persistent thinness vs severe early onset obesity and performed three-way association analyses: obese vs. thin, obese vs controls, controls vs. thin (Methods, S1 Table). After quality control, 41,266,535 variants remained for association analyses in the three cohorts: SCOOP vs STILTS, SCOOP vs UKHLS and UKHLS vs STILTS. Of the 97 established BMI associated loci from GIANT [24], we found that 40 were nominally significant (*p*<0.05) in SCOOP vs UKHLS and 15 in UKHLS vs STILTS (S2 Table). Direction of effect was consistent for all of these loci, which was more than expected by chance (binomial *p* = 9.09x10^-13^ and binomial *p* = 3.05x10^-5^, respectively). Overall, the proportion of phenotypic variance explained by the 97 established BMI associated loci was 10.67% in SCOOP vs UKHLS, and 4.33% in STILTS vs UKHLS (Methods). Evaluation of association results in thin (STILTS) and obese (SCOOP) individuals, compared to the same controls (UKHLS), suggested that the results are not a mirror image of each other (Figs 1–2), however we found little evidence of non-additive effects at the loci explaining this discrepancy (see below). We observed a striking difference in association results in the *FTO* locus where the lead intronic obesity risk variant, rs1558902, showed a moderate effect size and modest evidence of association in controls compared to thin individuals from STILTS (*p* = 0.00027, OR = 1.17, 95% CI [1.08,1.28], EAF = 0.39), despite having a large effect and being associated at genome-wide significance levels in SCOOP (*p* = 1.25x10^-17^, OR = 1.43, 95% CI [1.32,1.55], EAF = 0.41), and *GNAT2* also showed a larger effect and significance in the analysis of obese compared to control individuals (*p* = 1.26x10^-4^, OR = 1.57, 95% CI [1.25, 1.97], EAF = 0.03), than in the thin analysis (*p* = 0.52, OR = 1.10, 95% CI [0.82, 1.47], EAF = 0.02, Fig 1, S2 Table). This discrepancy in association strength and effect size was also seen at the opposite end of the BMI spectrum in *CADM2* where the lead SNP, rs13078960, showed evidence of association in STILTS (*p* = 9.48x10^-4^, OR = 1.2, 95% CI [1.08, 1.33], EAF = 0.20) but no association in SCOOP (*p*>0.05). In contrast to results at the *FTO* and *CADM2* loci, for *MC4R* the results are more comparable, with genome-wide significant association in obese individuals (rs6567160, *p* = 7.91x10^-9^, OR = 1.31, 95% CI [1.19, 1.43], EAF = 0.25) and highly significant association results in thin individuals (*p* = 1.38x10^-5^, OR = 1.26, 95% CI [1.13, 1.39], EAF = 0.23, S2 Table). To formally test if these results were significantly different from those expected under a model where loci act additively across the BMI distribution, we simulated 10,000 different populations of 1 million individuals with genotypes for the 97 established BMI loci using allele frequencies in the European population, and then simulated a phenotype using the effect sizes in GIANT (Methods). These simulations detected fourteen loci with nominally significant deviation from an additive model, however none remained significant after correction for the number of tests (*p* = 0.05/97*2 = ~0.0002, S3 Table), though *CADM2* was nominally significant in both SCOOP and STILTS analyses, with slightly lower OR detected in SCOOP compared to simulated data, and slightly higher OR detected in STILTS compared to simulated data (S3 Table). Recent work in mouse knockouts has shown *CADM2* plays an important role in systemic energy homeostasis [25] and variants near the gene have also been recently linked to habitual physical activity in humans [26]. Since SCOOP participants are significantly younger than UKHLS, we used summary statistics from a subset of the ALSPAC cohort [27] which consists of 4,964 children aged 13–16 to test if the observed OR differences in SCOOP vs UKHLS, compared to STILTS vs UKHLS, were due to age effects in SCOOP (Methods). For the 97 GIANT loci overall there were no significant differences in the ORs when comparing SCOOP to UKHLS or SCOOP to ALSPAC (z-test, p>0.05) except for rs2245368 (*PMS2L11* locus, z-test *p* = 3.81x10^-5^, S4 Table). In combination, these results suggest that the observed differences in ORs and p-values could have arisen because our severe obese cases are much more extreme (i.e. deviate more from the mean) than the healthy thin individuals, and that our obese and thin sample sizes gave us limited power to detect significant differences compared to the additive model.

Next we investigated the association of a genetic risk score, generated from the 97 BMI associated loci from GIANT [24] on BMI category (i.e. thin, normal, obese) using an ordinal logistic regression (Methods). As expected, the standardised BMI genetic risk score was strongly associated with BMI category (weighted score *p* = 8.59x10^-133^). We found that the effect of a one standard deviation increase in the standardised BMI genetic risk score was significantly larger for obese vs. (thin & normal) than for (obese & normal) vs. thin (*p* = 7.48x10^-11^, S1 Appendix) with odds ratio and 95% confidence intervals of 1.94 (1.83, 2.07) and 1.50 (1.42, 1.59) respectively. However, using the simulations described above (Methods), we confirm that the larger OR for obese vs. (thin & normal) is not significantly different (*p* = 0.41) than what we would expect given an additive genetic model, and the different degrees of extremeness in our thin and obese cases. Mean GRS in each BMI category was also not significantly different from that predicted via simulations (S1 Fig, Methods).

### Genetic Correlation between persistent thinness, severe early onset childhood obesity and BMI

Given the observed differences in association results from thin and obese individuals, compared to the same set of control individuals, we next explored the genetic correlation of severe early onset obesity, persistent thinness and BMI using LD score regression (Methods). For this, we used summary statistics from the SCOOP vs UKHLS, STILTS vs UKHLS and BMI data from participants in UK Biobank (UKBB, Methods). As expected from the association results, the genetic correlation of severe early onset obesity and BMI was high (r = 0.79, 95% CI [0.69, 0.89], *p* = 1.14x10^-52^). We also observed weaker negative correlation between persistent thinness and BMI (r = -0.69, 95% CI [-0.86, -0.51], *p* = 1.17x10^-14^), and between persistent thinness and severe obesity (r = -0.49, 95% CI [-0.17, -0.82], *p* = 0.003). As an inverse genetic correlation between BMI, obesity and anorexia nervosa (a disorder that is characterised by thinness and complex behavioural manifestations) has recently been reported [28], we also tested for genetic correlation with anorexia nervosa, and found that neither severe early onset obesity, nor persistent thinness, were significantly correlated with anorexia nervosa (r = -0.05, 95% CI [-0.15,0.05], *p* = 0.33 and r = 0.13, 95% CI [-0.02,0.28], *p* = 0.09, respectively; Methods).

### Association signals for persistent thinness and severe early onset obesity replicate established BMI associated loci

Given available genome-wide directly genotyped and imputed data we sought evidence for novel signals associated with either end of the BMI distribution (persistent thinness or severe early onset obesity; Methods) but found no novel replicating loci (details below). In all three discovery analyses, in addition to loci mapping to established BMI and obesity loci, we identified *PIGZ* and *C3orf38*, two putative novel loci in the thin vs control analysis, that reached conventional genome-wide significance (GWS) (*p*≤5x10^-8^) (S5–S7 Tables, Fig 2). However, an additional 125 SNPs, in 118 distinct loci, reached the arbitrary threshold of *p* ≤10^−5^ in at least one analysis, for which we sought replication (S5–S7 Tables).

As our obese and thin cases (SCOOP and STILTS) lie at the very extreme tails of the BMI distribution, there are few comparable replication datasets. We therefore used the UKBB dataset and selected individuals at the top (BMI> = 40, N = 7,526) and bottom end of the distribution (BMI≤19, N = 3,532) to more closely match the BMI criteria of our clinically ascertained thin and obese individuals. We used 20,720 samples from the rest of the UKBB cohort as a control set (Methods, S2 Fig). In cases where lead variants or proxies (r^2^>0.8) were not currently available in the full UKBB genetic release we used results from the interim release using 2,799 individuals with BMI> = 40, 1,212 with BMI< = 19 and 8,193 controls (Methods). We noted a significant negative genetic correlation for our obese replication cohort with anorexia nervosa (r = -0.24, 95% CI [-0.37,-0.11], *p* = 0.01) and a positive genetic correlation for our thin cohort (r = 0.49, 95% CI [0.22–0.76] *p* = 0.0003). We also observed significant genetic correlation between obesity in the discovery and replication cohorts (r = 0.84, 95% CI [0.65–1] *p* = 5.05x10^-17^) and between thinness in the discovery and replication cohorts (r = 0.62, 95% CI [0.20–1] *p* = 0.004).

To further increase power, we took advantage of publicly available summary statistics from the GIANT Extremes obesity meta-analysis [20], the EGG childhood obesity study [30], and our own previous study on non-overlapping SCOOP participants (SCOOP 2013) [31], as additional replication datasets. For SCOOP vs. STILTS we used the GIANT BMI tails meta-analysis results [20] (up to 7,962 cases/8,106 controls from the upper/lower 5th percentiles of the BMI trait distribution). For SCOOP vs. UKHLS we used the GIANT obesity class III summary statistics [20] (up to 2,896 cases with BMI ≥40kg/m^2^ vs 47,468 controls with BMI <25 kg/m^2^), the EGG childhood obesity study [30] (children with BMI ≥95th percentile of BMI vs 8,318 children with BMI <50th percentile of BMI) and SCOOP 2013 [31]. Fixed effect meta-analyses yielded genome-wide significant signals at well-known BMI associated loci in both the obese vs. thin, and obese vs. control analyses, and both the *PIGZ* and *C3orf38* loci identified at the discovery stage failed to replicate when combined with additional data (Table 1, S7 Table). However, the *SNRPC* locus described here (rs75398113), though not independent from the previously described *SNRPC*/*C6orf106* locus (rs205262, r^2^ = 0.29) [24], appears to be driving the previously reported association at this locus (rs205262 conditioned on rs75398113, *p*_*conditioned*_ = 0.7, S8 Table). Both SNPs are eQTLs for *C6or106* and *UHRF1BP1* in multiple tissues including brain and colon tissues on GTEx however neither of these are obvious biological candidates linked to energy homeostasis.

**Table 1:** GWAS results for SNPs meeting *p*<5x10^-8^ in all three analyses.

**Obese vs. thin**						**Discovery cohort**				**Replication cohorts**					**Combined analysis**		
**rsID**	**Nearest gene**	**Chr**.	**Position (bp)**	**EA**	**NEA**	**OR (95% CI)**	**P value**	**EAF Ob**	**EAF Th**	**Cohort**	**OR (95% CI)**	**P value**	**EAF Ob**	**EAF Th**	**OR (95% CI)**	**P value**	**HetPVal**
rs9930333	*FTO*	16	53799977	G	T	1.70(1.52,1.90)	2.30E-20	49.59%	37.46%	UKBB	1.46(1.38,1.55)	3.60E-36	48.26%	38.93%	1.48(1.42,1.54)	8.52E-76	3.34E-02
										GIANT^k^	1.43(1.34,1.54)	8.10E-25					
rs2168711	*MC4R*	18	57848531	C	T	1.66(1.45,1.89)	8.29E-14	28.90%	19.95%	UKBB	1.23(1.15,1.32)	2.19E-09	26.75%	22.90%	1.27(1.21,1.33)	2.02E-21	1.12E-04
										GIANT^k^	1.20(1.10,1.30)	1.80E-05					
rs6748821	*TMEM18*^a^	2	629601	G	A	1.65(1.42,1.91)	9.45E-11	86.69%	79.84%	UKBB	1.27(1.18,1.37)	1.31E-09	85.00%	81.69%	1.32(1.24,1.39)	7.76E-21	2.81E-03
										GIANT^k^	1.26(1.14,1.39)	9.90E-06					
rs506589	*SEC16B*	1	177894287	C	T	1.46(1.27,1.67)	5.42E-08	23.98%	18.07%	UKBB	1.25(1.17,1.35)	5.44E-10	23.11%	19.16%	1.28(1.21,1.35)	3.14E-20	1.21E-01
										GIANT^k^	1.25(1.14,1.37)	2.70E-06					
rs6738433	*ADCY3-DNAJC27*^b^	2	25159501	C	G	1.43(1.28,1.60)	1.71E-10	47.31%	43.92%	UKBB	1.21(1.14,1.28)	2.74E-10	50.70%	45.96%	1.19(1.14,1.24)	3.19E-17	6.25E-03
										GIANT^k^	1.10(1.03,1.17)	5.70E-03					
rs7132908	*FAIM2*	12	50263148	A	G	1.31(1.17,1.47)	2.26E-06	42.45%	36.27%	UKBB	1.18(1.11,1.25)	5.43E-08	41.11%	37.39%	1.20(1.15,1.26)	1.93E-16	2.52E-01
										GIANT^k^	1.20(1.10,1.30)	6.60E-06					
rs62107261	*FAM150B*	2	422144	T	C	2.37(1.75,3.20)	2.07E-08	96.37%	93.38%	UKBB	1.54(1.35,1.76)	3.57E-10	96.28%	94.36%	1.65(1.46,1.87)	1.15E-15	1.07E-02
rs12507026	*GNPDA2*^c^	4	45181334	T	A	1.30(1.17,1.46)	3.69E-06	47.29%	40.92%	UKBB	1.14(1.08,1.21)	8.76E-06	45.30%	41.98%	1.18(1.13,1.23)	5.53E-15	4.06E-02
										GIANT^k^	1.20(1.12,1.28)	3.10E-07					
rs75398113	*SNRPC*	6	34728071	C	A	1.53(1.27,1.85)	8.91E-06	11.95%	8.04%	UKBB	1.24(1.12,1.37)	2.07E-05	10.47%	8.52%	1.30(1.19,1.42)	5.19E-09	5.56E-02
rs13135092	*SLC39A8*	4	103198082	G	A	1.58(1.30,1.93)	4.70E-06	10.50%	7.24%	UKBB	1.25(1.12,1.39)	5.57E-05	9.24%	7.52%	1.32(1.20,1.45)	1.06E-08	3.59E-02
**Obese vs. controls**																	
**rsID**	**Nearest gene**	**Chr**.	**Position (bp)**	**EA**	**NEA**	**OR (95% CI)**	**P value**	**EAF Ob**	**EAF Co**	**Cohort**	**OR (95% CI)**	**P value**	**EAF Ob**	**EAF Co**	**OR (95% CI)**	**P value**	**HetPVal**
rs9928094	*FTO*	16	53799905	G	A	1.44(1.33,1.57)	1.42E-18	49.50%	41.32%	UKBB	1.30(1.25,1.35)	2.74E-41	48.34%	41.91%	1.32(1.29,1.36)	5.94E-101	4.41E-05
										SCOOP 2013	1.46(1.34,1.60)	4.88E-17					
										EGG	1.21(1.15,1.28)	7.20E-13					
										GIANT^l^	1.43(1.34,1.54)	6.60E-25					
rs35614134	*MC4R*^d^	18	57832856	AC	A	1.31(1.20,1.44)	6.27E-09	29.01%	23.69%	UKBB	1.22(1.16,1.27)	1.25E-18	26.72%	23.15%	1.23(1.20,1.27)	1.57E-43	3.55E-01
										SCOOP 2013	1.32(1.19,1.46)	1.22E-07					
										EGG	1.22(1.15,1.30)	1.27E-10					
										GIANT^l^	1.20(1.10,1.30)	1.70E-05					
rs66906321	*TMEM18*^e^	2	630070	C	T	1.40(1.24,1.57)	2.35E-08	85.78%	81.35%	UKBB	1.17(1.11,1.24)	3.44E-09	84.44%	82.20%	1.25(1.21,1.29)	9.72E-35	1.33E-02
										SCOOP 2013	1.39(1.24,1.57)	6.65E-08					
										EGG	1.28(1.19,1.37)	5.15E-12					
										GIANT^l^	1.27(1.15,1.40)	3.40E-06					
rs7132908	*FAIM2*^f^	12	50263148	A	G	1.22(1.12,1.32)	3.27E-06	42.45%	37.82%	UKBB	1.15(1.10,1.19)	5.37E-12	41.11%	37.71%	1.17(1.14,1.21)	2.38E-31	4.86E-01
										SCOOP 2013	1.23(1.12,1.35)	8.89E-06					
										EGG	1.18(1.11,1.25)	1.24E-08					
										GIANT^l^	1.20(1.10,1.30)	6.60E-06					
rs2384060	*ADCY3-DNAJC27*^g^	2	25135438	G	A	1.23(1.13,1.34)	1.53E-06	43.52%	38.90%	UKBB	1.11(1.07,1.15)	4.89E-08	47.67%	44.93%	1.14(1.11,1.17)	9.39E-23	1.13E-01
										SCOOP 2013	1.09(1.00,1.19)	5.01XE-02					
										EGG	1.18(1.12,1.24)	1.02E-09					
										GIANT^l^	1.12(1.04,1.19)	1.60E-03					
rs11209947	*NEGR1*^h^	1	72808551	A	T	1.30(1.17,1.44)	8.51E-07	76.58%	72.18%	UKBB	1.11(1.05,1.16)	4.53E-05	81.18%	79.76%	1.17(1.13,1.21)	5.17E-20	7.26E-05
										SCOOP 2013	1.46(1.30,1.63)	2.21E-10					
										EGG	1.13(1.06,1.22)	4.60E-04					
										GIANT^l^	1.22(1.11,1.35)	5.60E-05					
rs12735657	*SEC16B*^i^	1	177809133	C	T	1.24(1.13,1.37)	9.72E-06	24.26%	20.46%	UKBB	1.12(1.07,1.17)	1.48E-06	22.87%	20.94%	1.15(1.12,1.19)	7.26E-19	1.79E-01
										SCOOP 2013	1.20(1.07,1.33)	1.18E-03					
										EGG	1.14(1.06,1.21)	1.52E-04					
										GIANT^l^	1.22(1.11,1.34)	1.80E-05					
rs13104545	*GNPDA2*	4	45184907	A	G	1.27(1.15,1.40)	1.61E-06	27.41%	23.45%	UKBB	1.07(1.02,1.11)	5.35E-03	24.36%	23.26%	1.13(1.09,1.17)	1.47E-11	9.39E-05
										EGG	1.13(1.04,1.22)	3.39E-03					
										GIANT^l^	1.34(1.20,1.49)	1.20E-07					
rs112446794	***CEP120***^j^	5	122665465	T	C	1.23(1.13,1.35)	2.08E-06	33.15%	28.69%	UKBB	1.07(1.02,1.11)	2.55E-03	29.47%	28.21%	1.09(1.06,1.13)	3.45E-10	3.33E-02
										SCOOP 2013	1.08(0.98,1.19)	1.38E-01					
										EGG	1.12(1.06,1.18)	1.22E-04					
										GIANT^l^	1.05(0.97,1.13)	2.40E-01					
rs3760091	*SULT1A1*	16	28620800	C	G	1.24(1.14,1.35)	1.56E-06	64.89%	60.23%	UKBB	1.09(1.04,1.14)	1.19E-04	63.49%	61.44%	1.12(1.07,1.16)	2.65E-08	8.49E-03

EA = Effect allele (BMI increasing allele); NEA = Non-effect allele; OR = Odds ratio; 95% CI = 95% confidence interval for the odds ratio; EAF = effect allele frequency. Positions mapped to hg19, Build 37.

^a^rs12995480 used as proxy in GIANT.

^b^rs2384054 used as proxy in GIANT.

^c^rs12641981 used as proxy in GIANT.

^d^rs663129 used as proxy in GIANT, EGG and SCOOP 2013.

^e^rs13007080 used as proxy in GIANT, EGG and SCOOP 2013.

^f^rs7138803 used as proxy in SCOOP 2013.

^g^rs6722587 used as proxy in GIANT, EGG and SCOOP 2013.

^h^rs4132288 used as proxy in GIANT, EGG and SCOOP 2013.

^i^rs1460940 used as proxy in GIANT, EGG and SCOOP 2013.

^j^rs1366333 used as proxy in GIANT, EGG and SCOOP 2013.

^k^GIANT BMI tails [20].

^l^GIANT obesity class III [20].

Finally, we used the independent BMI dataset from UKBB (Methods) to investigate whether any of the loci meeting our arbitrary *p* ≤10^−5^ in discovery efforts, were independently associated with BMI as a continuous trait. This identified a novel BMI-associated locus near *PKHD1* (SCOOP vs. STILTS *p* = 5.99x10^-6^, SCOOP vs. UKHLS *p* = 2.13x10^-6^, BMI *p* = 2.3x10^-13^, S9 Table). Furthermore, we note that when comparing the signals we took for replication (based on case control analyses) with association results with BMI as a continuous trait derived from an independent set of samples from UKBB, there are more directionally consistent and nominally significant associations with BMI than expected by chance suggesting that amongst these loci, there may be additional real associations (binomial p = 4.88x10^-4^, and binomial p = 9.77x10^-3^, respectively, Methods, S9 Table).”

Despite the smaller sample size, the obese vs thin comparison had increased power to detect some loci (S3 Fig), including a recently discovered variant near *FAM150B* [32] (rs62107261, MAF = ~5%), which did not meet our p<10^−5^ threshold to be taken forward for replication in obese vs controls analysis (*p* = 2.36x10^-4^).

## Discussion

Here we present results from the largest to-date GWAS performed on healthy individuals with persistent thinness and provide the first insights into the genetic architecture of this trait. To our knowledge, there are only two other studies using thin individuals with comparable mean BMIs [21,22]. The study by Hinney *et al*. [21] (N = 442), was only able to detect *FTO* at genome-wide significance level with rs1121980 having a similar effect to that which we report (OR = 1.66 vs OR = 1.69 in our data). In the Scannell Bryan *et al*. [22] study, Bangladeshi individuals were reportedly thin and malnourished, and a single suggestive association was found with an intronic variant in *NRXN3* (rs12882679, p = 9.57x10^-7^) which is not significant in our study (p = 0.77).

Using genome-wide genotype data we show that persistent healthy thinness, similar to severe obesity (h^2^ = 32.33%), is a heritable trait (h^2^ = 28.07%). Persistent healthy thinness and severe childhood obesity are negatively correlated (r = -0.49, 95% CI [-0.17, -0.82], *p* = 0.003), and share a number of genetic risk loci. Nonetheless, the genetic overlap between the two clinically ascertained traits appears to be incomplete, as highlighted by some loci which were more strongly associated at one end of the BMI distribution (e.g. *CADM2*), while others, appeared to exert effects across the entire BMI spectrum (e.g. *MC4R* [9,33,34]). Further exploration by simulation demonstrated that these differences are likely to be due to the different degrees of extremeness of the two clinical cohorts (i.e. a similar degree of thinness to that of the obese cohort may not be compatible with healthy human life) and not due to a deviation from additive effects of the tested loci on BMI, with the possible exception of *CADM2* which deviated from expectation with nominal significance in both the obese and the thin analysis (S3 Table). This is in contrast with earlier studies which suggested larger effects at the higher end of the BMI distribution [35,36] but in agreement with more recent observations contrasting the bottom 5% and top 5% of the BMI tails where associated loci were also consistent with additive effects [20]. This is also in contrast with a previous study on height, where a deviation from additivity was found, but only for short individuals in the bottom 1.5% of the distribution [37], which suggests that analysis focused just on the most extreme individuals may be warranted.

Focusing on the 97 previously established BMI associated loci [24], we show that the percentage of phenotypic variance explained by these loci is lower in persistently thin (4.33%) compared to obese individuals (10.67%), and that the effect of an increase/decrease in the BMI genetic risk score was much larger, on average, for obese individuals than for thin individuals (one standard deviation increase in the standardised BMI genetic risk score of 1.94, 95% CI (1.83, 2.07) and 1.50, 95% CI (1.42, 1.59), respectively) which is consistent with the difference in BMI units amongst categories. And, although our analysis using age-matched controls from ALSPAC suggested that the observed differences in ORs, comparing obese vs control individuals to controls vs thin individuals, was unlikely to be due to age effects, we cannot completely exclude the possibility that different effects of age and sex in our discovery cohorts (S1 Table), and gene-by-environment interactions, could be influencing some of the results we observe. For example, gene-by-environment interactions and age effects have been previously reported at the *FTO* locus [38–41] where a larger effect is detected in younger adults. It is worth noting though that non-additive effects have also been observed in the *FTO* locus [42].

In studying thin individuals there are often concerns regarding the prevalence of eating disorders, notably anorexia nervosa amongst participants. We sought to carefully exclude eating disorders at two phases of recruitment (by medical history and by questionnaire). Additionally, we demonstrate that in our cohort of healthy thin individuals, anorexia nervosa is unlikely to be a confounder as the two traits are genetically only weakly correlated (r = 0.13, 95% CI [-0.02,0.28], *p* = 0.09). This was not the case for the UKBB replication cohort where a positive genetic correlation was observed (r = 0.49 95% CI [0.22–0.76] *p* = 0.0003). The positive genetic correlation with anorexia was still observed after removing individuals with medical conditions that could explain their low BMI (r = 0.62, 95% CI [0.30,0.92], *p* = 0.0001, Methods**)**. These results highlight the importance of the careful phenotyping performed in the recruitment phase and the utility of the STILTS cohort as a resource to study healthy and persistent thinness.

In the genome-wide association analyses amongst the signals we took forward for replication, in addition to detecting established BMI-associated loci, we find a novel BMI-association at *PKHD1* in the UKBB BMI dataset (rs10456655, β = 0.10, p = 2.3x10^-13^, S9 Table), where a proxy for this variant (rs2579994, r^2^ = 1 in 1000G Phase 3 CEU) has been previously nominally associated with waist and hip circumference (*p* = 5.60x10^-5^ and *p* = 4.40x10^-4^ respectively) [43]. In addition, we found associations at loci that have only recently been established using very large sample sizes. *FAM150B*, was only suggestively associated at discovery stage in Tachmazidou *et al*. (2017) [32] (n = 47,476, *p* = 2.57×10^−5^) whereas it reached genome-wide significance when contrasting SCOOP vs STILTS (n = 2,927, p = 2.07x10^-8^, S5 Table). Also, *PRDM6-CEP120* [5] was recently discovered in a Japanese study with a sample size of 173,430 and has not been previously reported in a European population. In our study, a signal near the locus (rs112446794, r^2^ = 0.36) showed suggestive evidence of association in SCOOP vs UKHLS (*p* = 2.08x10^-6^, S6 Table) with a significantly smaller sample size. Conditional analysis reveals the lead SNP in this study drives the association of the previously established signal (S8 Table). *CEP120* codes for centrosomal protein 120. Variants near this locus have been previously associated with height [44] and waist circumference in East Asians [45]. Missense variants in the gene itself have been associated with rare ciliopathies [46,47]. Lastly, amongst the signals we took for replication, and after removing known and newly established loci, we still observe an enrichment of directionally consistent and nominal associations in the analysis of BMI as a continuous trait, suggesting that some of these results may warrant additional investigation, in particular in similarly ascertained thin and obese cohorts. One such example is rs4447506, near *PIK3C3*, which was not only nominally significant and consistent in the independent UKBB BMI analysis (*p* = 1.5x10^-6^, S9 Table), but also in the Locke *et al*. (2015) [24] BMI results (*p* = 0.01), and in the GIANT BMI tails analysis we used as replication (S5 Table). We also note, that despite not reaching genome-wide significance in our discovery cohorts, we observe directionally consistent suggestive associations at a number of loci previously associated with BMI tails and with different obesity classes [20] (S10 Table). Altogether, these results highlight some power advantages of using clinically ascertained extremes of the phenotype distribution to detect associations and suggest that healthy thinness falls at the lower end of the polygenic BMI spectrum. It is worth noting though that these clinically ascertained extremes display evidence of incomplete genetic correlation with BMI, in contrast to previously described obesity classes (S4 Fig), so it is plausible that additional loci might be uncovered by focusing on clinical extremes.

As our results were based on clinically ascertained participants which met very specific criteria, it is worth noting these conclusions cannot be straightforwardly extrapolated to the general population. Experiments in animals have identified loci/genes associated with thinness/decreased body weight due to reduced food intake/increased energy expenditure/resistance to high fat diet-induced obesity [48,49], mechanisms that we hypothesise may contribute to human thinness. The STILTS cohort, being uncorrelated to anorexia nervosa, is an excellent resource in which to conduct such additional genetic exploration. Further genetic and phenotypic studies focused on persistently thin individuals may provide new insights into the mechanisms regulating human energy balance and may uncover potential anti-obesity drug targets.

## Methods

### Ethics statement

The study was reviewed and approved by the South Cambridgeshire Research Ethics Committee (12/EE/0172). All participants provided written informed consent prior to inclusion.

### Cohorts

SCOOP, STILTS and UKHLS cohorts were used for the heritability, genetic correlation, genetic risk score and association analyses with established BMI loci, as well as, used as a discovery cohort in the genome-wide association study (GWAS) and gene-based tests. UK Biobank samples were used for genetic correlation analysis and in the replication stages of the GWAS and gene-based tests. ALSPAC was used as an additional control dataset to UKHLS for comparison against SCOOP in the established BMI loci analysis.

#### STILTS

The aim was to recruit a new cohort of UK European people who are thin (defined as a body mass index < 18kg/m^2^) and well. After ethical committee approval (12/EE/0172), we worked with the NIHR Primary Care Research Network (PCRN) to collaborate with 601 GP practices in England. Each practice searched their electronic health records using our inclusion criteria (age 18–65 years, BMI<18 kg/m^2^) and exclusion criteria (medical conditions that could potentially affect weight (chronic renal, liver, gastrointestinal problems, metabolic and psychiatric disease, known eating disorders). A small number of individuals (n = 43) with a BMI of 19.0 kg/m^2^ were included as they had a strong family history of thinness. The case notes of each potential participant were reviewed by the GP or a senior nurse with clinical knowledge of the participant to exclude other potential causes of low body weight in discussion with the study team. Through this approach we identified 25,000 individuals who fitted our criteria for inclusion in the study. These individuals were invited to participate in the study; approximately 12% (2,900) replied consenting to take part. We obtained a detailed medical and medication history, screened for eating disorders using a questionnaire (SCOFF) that has been validated against more formal clinical assessment [50]. We excluded all participants who stated that they exercised every day/more than 3 times a week/whose reported activity exceeded 6 metabolic equivalents (METs) for any duration or frequency (http://www.who.int/dietphysicalactivity/physical_activity_intensity/en/). With these rather strict criteria for exercise, we sought to limit the contribution of exercise as a contributor to the thinness of participants in the STILTS cohort. We excluded people who were thin only at a certain point in their lives (often as young adults) to focus on those who were persistently thin/always thin throughout life as we hypothesised that this group would be enriched for genetic factors contributing to their thinness. We asked a specific question to identify these individuals: “have you always been thin?” Only those who answered positively were included. Questionnaires were manually checked by senior clinical staff for these parameters and for reported ethnicity (non-European ancestry excluded). DNA was extracted from salivary samples obtained from these individuals using the Oragene 500 kit according to manufacturer’s instructions (S1 Table).

#### SCOOP

With ethical committee approval (MREC 97/5/21), we have recruited 7,000 individuals with severe early-onset obesity (BMI standard deviation score (SDS) > 3; onset of obesity before the age of 10 years) to the Genetics of Obesity Study (GOOS) [51]. The Severe Childhood Onset Obesity Project (SCOOP) cohort [31] is a sub-cohort of GOOS comprised of ~4,800 British individuals of European ancestry; S1 Table). SCOOP individuals likely to have congenital leptin deficiency, a treatable cause of severe obesity, were excluded by measurement of serum leptin, and individuals with mutations in the melanocortin 4 receptor gene (*MC4R*) (the most common genetic form of penetrant obesity) were excluded by prior Sanger sequencing.

#### UKHLS

Understanding Society (UKHLS) is a longitudinal household study designed to capture economic, social and health information from UK individuals [52]. A subset of 10,484 individuals was selected for genome-wide array genotyping. This cohort was used as a control dataset with SCOOP and STILTS cases (S1 Table).

#### UK BIOBANK (UKBB)

This study includes approximately 487,411 participants with genetic data released (including ~50,000 from the UKBiLEVE cohort [53]) of the total 502,648 individuals from UK BioBank (UKBB). UKBB samples were genotyped on the UK Biobank Axiom array at the Affymetrix Research Services Laboratory in Santa Clara, California, USA and imputed to the Haplotype Reference Consortium (HRC) panel [54]. UKBiLEVE samples were genotyped on the UK BiLEVE array which is a previous version of the UK Biobank Axiom array sharing over 95% of the markers. To date, 487,411 samples with directly genotyped and imputed data are available and data was downloaded using tools provided by UK Biobank. Extensive data from health and lifestyle questionnaires is currently available as well as linked clinical records. BMI, as well as other physical measurements were taken on attendance of recruitment centre. Severely obese participants in the available data were defined as those with BMI ≥ 40 kg/m^2^ (N = 9,706) and thin individuals were defined as those with BMI ≤ 19 kg/m^2^ (N = 4,538). Given that it has been previously shown that type I error rate for variants with a low minor allele count (MAC) is inadequately controlled for in very unbalanced case-control scenarios [55], we randomly subsampled 35,000 individuals from the original 487,411 genotyped individuals and removed those with BMI≤19 or BMI ≥30, to generate an independent control set. The 25,856 participants remaining after BMI exclusions from the tails, generated a non-extreme set of individuals kept as putative controls (S2 Fig). The other 452,411 genotyped samples were kept as the BMI dataset for downstream analyses (S11 Table, S2 Fig). An interim release consisting of a subset 152,249 individuals from UKBB was released in May 2015. This interim release was imputed to a combined UK10K and 1000G Phase 3 reference panel and contains several variants which are not currently present in the HRC panel, as such it was used in some of the analyses described.

#### ALSPAC

The Avon Longitudinal Study of Parents and Children (ALSPAC) [27,56], also known as Children of the 90s, is a prospective population-based British birth cohort study. Ethical approval for the study was obtained from the ALSPAC Ethics and Law Committee and the Local Research Ethics Committees. Please note that the study website contains details of all the data that is available through a fully searchable data dictionary (http://www.bris.ac.uk/alspac/researchers/data-access/data-dictionary/). Further information about this cohort, including details of the genotyping and imputation procedures, can be found in S2 Appendix. This analysis was restricted to a subset of unrelated (identity-by-state < 0.05 [57]) children with genetic data and BMI measured between the age of 12 and 17 years (n = 4,964, 48.5% male). The mean age of the children was 14 years and the mean BMI 20.5.

### Genotyping and quality control

#### SCOOP, STILTS and UKHLS

For the SCOOP cohort, DNA was extracted from whole blood as previously described [31]. For the STILTS cohort, DNA was extracted from saliva using the Oragene saliva DNA kits (online protocol) and quantified using Qubit. All samples from SCOOP, STILTS and UKHLS were typed across 30 SNPs on the Sequenom platform (Sequenom Inc. California, USA) for sample quality control. Of the 3,607 SCOOP and STILTS samples submitted for Sequenom genotyping, 3,280 passed quality controls filters (90.9% pass rate). Of the 10,433 UKHLS samples, 9,965 passed Sequenom sample quality control (95.5% pass rate). Subsequently, UKHLS controls were genotyped on the Illumina HumanCoreExome-12v1-0 Beadchip. The 3,280 SCOOP and STILTS samples, and 48 overlapping UKHLS samples (to test for possible array version effects) were genotyped on the Illumina HumanCoreExome-12v1-1 Beadchip by the Genotyping Facility at the Wellcome Sanger Institute (WSI). Genotype calling was performed centrally for all batches at the WSI using GenCall. Criteria for excluding samples were as follows: i) concordance against Sequenom genotypes <90%; ii) for each pair of sample duplicates, exclude one with highest missingness; iii) sex inferred from genetic data different from stated sex; iv) sample call rate <95%; v) sample autosome heterozygosity rate >3 SDS from mean done separately for low (<1%) and high MAF(>1%) bins; vi) magnitude of intensity signal in both channels <90%; and vii) for each pair of related individuals (proportion of IBD (PI_HAT) >0.05), the individual with the lowest call rate was excluded. We performed SNP QC using PLINK v1.07 [58]. Criteria for excluding SNPs was: i) Hardy-Weinberg equilibrium (HWE) p<1x10^-6^; ii) Call rate <95% for MAF≥5%, call rate <97% for 1% ≤MAF<5%, and call rate <99% for MAF <1%. SMARTPCA v10210 [59] was used for principal component analysis (PCA). To verify the absence of array version effects we used PCA on the subset of shared controls genotyped on both versions of the array. Cut-offs for samples that diverged from the European cluster were chosen manually after inspecting the PCA plot. SNPs with discordant MAFs in the different versions of the array were excluded. After removal of non-European samples and 13 samples due to cryptic relatedness, 1,456 SCOOP and 1,471 STILTS samples remained for analysis. For UKHLS, 82 samples were removed after applying a strict European filter and 680 related samples were removed after applying a “3^rd^ degree” kinship filter in KING [60]. A total of 9,203 samples remained, of which 6,460 had a BMI >19 and <30 (“controls”).

#### UK BIOBANK

Sample QC was performed using all 487,411 samples. Criteria for excluding samples were as follows: i) supplied and genetically inferred sex mismatches; ii) heterozygosity and missingness outliers according to centrally provided sample QC files; iii) samples not used in kinship estimation by UKBB; iv) individuals that did not identify as “white british” or did not cluster with other “white british” in PCA analysis; v) samples that withdrew consent and vi) for each pair of related individuals (KING kinship estimate>0.0442), we randomly selected an individual preferentially keeping cases if one related individual is a control. After sample QC, thirteen individuals with underlying health conditions that could influence their BMI were also removed, twelve had BMI<14, and one had BMI>74. In the end, 7,526 obese, 3,532 thin and 20,720 non-extreme controls remained for case-control analyses. In addition, 387,164 samples remained for analysis of BMI as a continuous trait. There is an overlap of 10, 282 samples (~2.6% of the BMI dataset) with obese and thin cases (S2 Fig). The same procedure was performed on the interim release of 152,249 UKBB samples to produce a set of 2,799 obese, 1,212 thin, 8,193 controls and 127,672 individuals for the independent BMI dataset. All subsequent analyses on UKBB were also performed on this subset to query variants that are not currently available in the full UKBB release.

### Imputation and genome wide association analyses

#### SCOOP, STILTS and UKHLS single-variant association analysis

Genotypes from SCOOP, STILTS and UKHLS controls were phased together with SHAPEITv2 [61], and subsequently imputed with IMPUTE2 [62,63] to the merged UK10K and 1000G Phase 3 reference panel [64], containing ~91.3 million autosomal and chromosome X sites, from 6,285 samples. More than 98% of variants with MAF ≥0.5% had an imputation quality score of r^2^≥0.4, however variants with MAF <0.1% had a poor imputation quality with only 27% variants with r^2^≥0.4 (S5 Fig). First-pass single-variant association tests were done for all variants irrespective of MAF, or imputation quality score (see below). Analyses of 1,456 SCOOP, 1,471 STILTS and 6,460 controls (BMI range 19–30) of European ancestry were based on the frequentist association test, using the EM algorithm, as implemented in SNPTEST v2.5 [65], under an additive model and adjusting for six PCs and sex as covariates.

#### UKBB BMI dataset single-variant association analysis

For the BMI dataset, we used BOLT-LMM [66] to perform an association analysis with BMI using sex, age, 10 PCs and UKBB genotyping array as covariates.

#### Heritability estimates and genetic correlation

Summary statistics from the SCOOP vs. UKHLS, STILTS vs. UKHLS, UKBB obese vs controls, UKBB thin vs controls and UKBB BMI analyses were filtered and a subset of 1,197,969 HapMap3 SNPs was kept in each dataset. Using LD score regression [67] we first calculated the heritability of severe childhood obesity (SCOOP vs UKHLS) and persistent thinness (STILTS vs UKHLS). For severe childhood obesity, we estimated a prevalence of 0.15% using the BMI centile equivalent to 3SDS in children [68]. In the case of persistent thinness (BMI< = 19), we used a GP based cohort for our prevalence estimates: CALIBER [69]. The CALIBER database consists of 1,173,863 records derived from GP practices. For the heritability analysis, we used a prevalence estimate of 2.8% for BMI< = 19 (Claudia Langenberg and Harry Hemingway, personal communication). We also used LD score regression to calculate the genetic correlation of SCOOP with STILTS, SCOOP with UKBB obese, SCOOP with BMI, STILTS with UKBB thin and STILTS with BMI. The genetic correlation between obesity and persistent thinness with anorexia was estimated using the summary statistics from SCOOP vs UKHLS and STILTS vs. UKHLS, and summary statistics available from the Genetic Consortium for Anorexia Nervosa (GCAN) in LD Hub [70]. The same analysis was repeated for UKBB obese vs controls and UKBB thin vs controls. Genetic correlation estimates for BMI vs Overweight, Obesity Class 1, Obesity Class 2 and Obesity Class 3 were also extracted from LD Hub (S4 Fig).

#### Comparison with established GIANT BMI associated loci

We obtained the list of 97 established BMI associated loci from the publicly available data from the GIANT consortium [24]. We used this list as we wanted to focus on established common variation in Europeans with accurate effect sizes for simulations. In order to test whether there is evidence of enrichment of nominally significant signals with consistent direction of effect, we performed a binomial test using the subset of signals with nominal significance in the SCOOP vs UKHLS, and STILTS vs UKHLS analyses. Variance explained was calculated using the rms package [71] v4.5.0 in R [72] and Nagelkerke’s R^2^ is reported. Power calculations were performed using Quanto [73]. To calculate ORs and SE from the ALSPAC BMI summary statistics we used genotype counts from SNPTEST output. We then used a z-test to test for significant differences between the OR calculated using genotype counts of SCOOP and ALSPAC against the SCOOP vs. UKHLS OR.

#### Simulations under an additive model

We created 10,000 simulations of 1 million individuals for the 97 GIANT BMI loci randomly sampling alleles based on the allele frequency from the sex-combined European dataset reported in Locke *et al*. [24] using an R script. For each simulated genotype, we simulated phenotypes with DISSECT [74] using the effect size in GIANT and then removed all samples from the lower tail where the phenotype was <3SDs to better reproduce the actual BMI distribution. Afterwards we randomly sampled 1,471 individuals from the bottom 2.8% and 1,456 from top 0.15% and compared against a random set of 6,460 controls from the equivalent percentiles to BMI 19–30. Finally, for each of these loci, we calculated the absolute difference between our observed OR and the mean OR from the simulations and counted how many times we saw an equal or larger absolute difference in the simulated data and assigned a p-value. This was done separately for SCOOP vs UKHLS and STILTS vs UKHLS.

#### Genetic risk score

The R package GTX (https://cran.r-project.org/web/packages/gtx/index.html) was used to transpose genotype probabilities into dosages, and a combined dosage score, weighted by the effect size from GIANT, for 97 BMI SNPs [24] was calculated and standardised. We checked whether there was an ordinal relationship between the genetic risk score and BMI category (i.e. thin, normal, or obese) using ordinal logistic regression with the clm function in the ordinal R package. While the assumption of equal variance appears to hold (S6 Fig), the proportional odds assumption indicating equal odds between thin, normal, and obese groups is violated for the BMI genetic risk score and some of the principal component covariates (i.e., PC2, PC3, and PC6). As our primary model, we ran a partial proportional odds model adjusting for PC1, PC4, and PC5 and allowing the BMI genetic score, PC2, PC3, and PC6 to vary between BMI category. To check for consistency, we ran a partial proportional odds model adjusting for the first six PCs and allowing only the BMI genetic score to vary between BMI group and a full proportional odds model allowing all six PCs and the BMI genetic score to vary between BMI group (S1 Appendix). Using ANOVA, we formally tested the proportional odds assumption for the BMI genetic risk score. A genetic risk score was created and an ordinal logistic regression was run for each of the 10,000 simulations. We compared the observed test statistic testing whether the odds were the same by BMI category to the 10,000 simulation test statistics. We calculated the p-value as the number of simulations with a test statistic larger than that observed in the real data. A mean genetic risk score was also calculated for each BMI category (obese, thin and controls) across the 10,000 simulations. A t-test was used to test whether the mean observed GRS score in each category was significantly different from the one estimated using the simulations.

#### Discovery stage GWAS

First pass single-variant association analyses results were used as discovery datasets for the GWAS. After association analysis, we removed variants with MAF<0.5%, an INFO score <0.4, and HWE p<1x10^-6^, as these highlighted regions of the genome that were problematic, including CNV regions with poor imputation quality. Quantile-quantile plots indicated that the genomic inflation was well controlled for in SCOOP-UKHLS (λ = 1.06) and STILTS-UKHLS (λ = 1.04), and slightly higher for SCOOP-STILTS (λ = 1.08, S7 Fig). We used LD score regression [67] to correct for inflation not due to polygenicity. To identify distinct loci, we performed clumping as implemented in PLINK [58] using summary statistics from the association tests and LD information from the imputed data, clumping variants 250kb away from an index variant and with an r^2^>0.1. In order to further identify a set of likely independent signals we performed conditional analysis of the lead SNPs in SNPTEST to take into account long-range LD. A total of 135 autosomal variants with p<1x10^-5^ in any of the three case-control analyses were taken forward for replication in UKBB. All case-control results are reported with the lower BMI group as reference.

#### UKBB association analysis

We tested 1,208,692 SNPs for association under an additive model in SNPTEST using sex, age, 10 PCs and UKBB genotyping array as covariates. Three comparisons were done: obese vs thin, obese vs controls and controls vs thin. Variants with an INFO score <0.4, HWE *p*<1x10^-6^ were filtered out from the results. Inflation factors were calculated using HapMap markers. The LD score regression intercepts were 1.0074 in obese vs thin, 1.0057 in obese vs controls and 1.009 in thin vs controls. We used all thin individuals, regardless of health status, as our replication cohort to maximize power. However, using ICD10 codes and self-reported illness data (S12 and S13 Tables) to remove individuals who had a relevant medical diagnosis before date of attendance at UKBB recruitment centre, yielded 2,518 thin individuals and materially equivalent results (S8 Fig).

#### GIANT, EGG and SCOOP 2013 summary statistics

We obtained summary statistics for the GIANT Extremes obesity meta-analysis [20] from http://portals.broadinstitute.org/collaboration/giant/index.php/GIANT_consortium_data_files. Summary statistics for EGG [30] were obtained from http://egg-consortium.org/childhood-obesity.html. We used summary statistics from our previous study of 1,509 early-onset obesity SCOOP cases compared to 5,380 publicly available WTCCC2 controls (SCOOP 2013) [31]. Data for the SCOOP cases is available to download from the European Genome-Phenome Archive (EGA) using accession number EGAD00010000594. The control samples are available to download using accession numbers EGAD00000000021 and EGAD00000000023. These replication studies are largely non-overlapping with our discovery datasets and each-other. When a lead variant was not available in a replication cohort, a proxy (r^2^≥ 0.8) was used in the meta-analysis.

#### Replication meta-analysis

We meta-analysed summary statistics for the 135 variants reaching *p*<1x10^-5^ in SCOOP/STILTS/UKHLS with the corresponding results from UKBB and study specific replication cohorts (S5–S7 Tables). For obese vs. thin and obese vs. controls comparisons we used fixed-effects meta-analysis correcting for unknown sample overlap in replication cohorts using METACARPA [75]. For thin vs. controls we used a fixed-effects meta-analysis in METAL [76]. Heterogeneity was assessed using Cochran’s Q-test heterogeneity p-value in METAL. A signal was considered to replicate if it met all the following criteria: i) consistent direction of effect; ii) p<0.05 in at least one replication cohort; and iii) the meta-analysis p-value reached standard genome-wide significance (p<5x10^-8^). Given that we are querying additional variants on the lower allele frequency spectrum, one could also use a more strict genome-wide significance threshold taking into account the increased number of tests (*p*≤1.17x10^-8^) [77]. In practice, this only affected one previously established signal (*SULT1A1*, rs3760091) in our obese vs. controls analysis that fell just below this threshold (S6 Table). rs4440960 was later removed from final results (SCOOP vs UKHLS and STILTS vs UKHLS) after close examination revealed it was present in a CNV region with poor imputation quality.

#### Comparison of newly established candidate loci and UKBB independent BMI dataset

We identified eleven signals in SCOOP vs STILTS, nine in SCOOP vs UKHLS and two in UKHLS vs STILTS that were nominally significant in the UKBB BMI dataset GWAS, and directionally consistent. A binomial test was used to check for enrichment of signals with consistent direction of effect (S9 Table).

#### Lookup of previously identified obesity-related signals in our discovery datasets

We took all signals reaching genome-wide significance, or identified for the first time in the GIANT Extremes obesity meta-analysis [20], with either the tails of BMI or obesity classes, and in childhood obesity studies [30,31] and performed look-up of those signals in all three of our discovery analyses (SCOOP vs STILTS, SCOOP vs UKHLS and UKHLS vs STILTS). ORs and p-values from the previous studies and look-up results from our discovery datasets are reported in S10 Table.

## Supporting information

S1 Appendix Assessing equal vs. unequal effects for the genetic risk score. (DOCX)

S2 Appendix The Avon Longitudinal Study of Parents and Children. (DOCX)

S1 Fig Mean GRS for SCOOP and STILTS compared to simulations. Histogram represents mean GRS scores for each BMI category across 10,000 simulations. Vertical red line highlights the observed value in real data. p = p-value of difference.
(TIF)

S2 Fig Summary of the UKBB sample sets after QC. Venn Diagram showing sample numbers and overlap between UKBB sample sets used in genetic correlation (BMI dataset) and GWAS replication (obese, controls, thin) analyses.
(TIF)

S3 Fig Manhattan plot of SCOOP vs STILTS. Manhattan plot produced in EasyStrata, red line indicates genome-wide significance threshold at p = 5x10-08. Orange line indicates discovery significance threshold at p = 1x10-05. Black labels highlight known BMI/obesity loci that were taken forward for replication and yellow peaks indicate those that met genome-wide significance after replication.
(TIF)

S4 Fig Genetic correlation of traits and BMI. Genetic correlation estimates and 95% CI for severe early-onset childhood obesity (SCOOP), healthy persistent thinness (STILTS), Obesity Class 3, Obesity Class 2, Obesity Class 1 and Overweight. Dotted lines represent complete genetic correlation.
(TIF)

S5 Fig Quality of UK10K+1000G imputed genotypes. Percentage of variants with INFO score (r^2^)>0.4, as derived from the IMPUTE2 imputation algorithm, stratified by minor allele frequency across all samples (SCOOP, STILTS and UKHLS).
(TIF)

S6 Fig Box and density plots of risk score weighted by effect size for 97 BMI associated SNPs from GIANT. A weighted genetic risk score for each individual was obtained by summing genotype dosages multiplied by the effect (beta) estimates from GIANT for each of the 97 SNPs. To check the equal variance assumption, we used a box plot (left) and density plot (right). Density plot: Green = STILTS; Blue = UKHLS; Red = SCOOP.
(TIF)

S7 Fig Quantile-quantile plots of three discovery analysis cohorts. Q-Q plots of LD Score Regression-corrected p-values for the three analysis cohorts used for the discovery analysis, produced in EasyStrata. Red = SCOOP vs. STILTS; Black = SCOOP vs. UKHLS, Blue = STILTS vs. UKHLS. Variants passing QC and with MAF > = 0.5% are shown. LD Score regression intercept (λLD) values before correction are shown for each analysis.
(TIF)

S8 Fig Quantile-quantile plots for UKBB case-control analysis with different exclusion criteria for thin individuals. Q-Q plot using all thin individuals as cases (Full UKBB) and removing individuals based on ICD10 and self-reported data (ICD10+self-reported filter). Correlation for–log10 p-values is shown (r = 0.7462).
(TIF)

S1 Table Summary of discovery sample sets. (XLSX)

S2 Table 97 BMI SNPs from the GIANT consortium study and their summary statistics in our three analysis cohorts. (XLSX)

S3 Table Nominally significant loci for non-additive effect in extremes. (XLSX)

S4 Table Difference in SCOOP OR when using ALSPAC as control dataset vs. UKHLS. (XLSX)

S5 Table Discovery, replication and meta-analysis results for 32 SNPs meeting P<10–5 in discovery association results of SCOOP vs STILTS analysis. (XLSX)

S6 Table Discovery, replication and meta-analysis results for 66 SNPs meeting P<10–5 in discovery association results of SCOOP vs UKHLS analysis. (XLSX)

S7 Table Discovery, replication and meta-analysis results for 37 SNPs meeting P<10–5 in discovery association results of UKHLS vs STILTS analysis. (XLSX)

S8 Table Reciprocal analysis of previously established signals and lead signals in this study. (XLSX)

S9 Table Consistency of the direction of effect in candidate loci meeting p<1x10-5 in the discovery stages with BMI dataset GWAS. (XLSX)

S10 Table Published loci from GIANT, EGG and SCOOP 2013 not reaching genome-wide significance in our study. (XLSX)

S11 Table Summary of UKBB sample sets. (XLSX)

S12 Table ICD10 codes used to exclude thin individuals in UKBB. (XLSX)

S13 Table Self-reported illness codes used to exclude thin individuals in UKBB. (XLSX)
