# Supplementary material for: Genetic architecture of human thinness compared to severe obesity
Source: PLoS Genet. 2019 Jan 24;15(1):e1007603. doi: 10.1371/journal.pgen.1007603 (PMC6345421; doi:10.1371/journal.pgen.1007603)
Supplement: S1 Appendix — (DOCX) [file pgen.1007603.s001.docx]

**S1 Appendix. Assessing equal vs. unequal effects for the genetic risk score.**

We compared nested models using Analysis of Variance (ANOVA) to formally assess the proportional odds assumption of whether allowing the effect size of the odds ratio to differ between group is a better model than constraining the effect sizes to be equal. We assessed allowing the odds ratios to vary by obesity group for the genetic risk score while controlling for the first six principal components (PCs). PC2, PC3, and PC6 were found to produce a significantly better model when allowed to vary by obesity category. Thus, our primary model was a partial proportional odds model adjusting for the first six PCs allowing the odds ratios for PC2, PC3, and PC6 to vary by obesity group and constraining PC1, PC4, and PC5 to have equal odds ratios by obesity group. In this primary model, the model allowing the odds ratio of the genetic risk score to vary by obesity group was significantly better than constraining the odds ratio to be equal (p=7.48x10^-11^). To ensure that this result was robust to how we were constraining or not constraining the PCs, we repeated the test allowing the effect sizes for all six PCs to vary by obesity group (PC-unconstrained) and forcing the effect sizes for all six PCs to be equal over obesity groups (PC-constrained). Similar to the primary model, allowing the odds ratio for the genetic risk score to vary by obesity group was a significantly better model in both the PC-unconstrained and PC-constrained models (p=8.47 x10^-11^ and p=7.82x10^-11^ respectively).
