# Supplementary material for: Genetic architecture of human thinness compared to severe obesity
Source: PLoS Genet. 2019 Jan 24;15(1):e1007603. doi: 10.1371/journal.pgen.1007603 (PMC6345421; doi:10.1371/journal.pgen.1007603)
Supplement: S2 Appendix — (DOCX) [file pgen.1007603.s002.docx]

**S2 Appendix. The Avon Longitudinal Study of Parents and Children**

*ALSPAC: Description of study numbers*

ALSPAC recruited 14,541 pregnant women resident in Avon, UK with expected dates of delivery 1st April 1991 to 31st December 1992. 14,541 is the initial number of pregnancies for which the mother enrolled in the ALSPAC study and had either returned at least one questionnaire or attended a “Children in Focus” clinic by 19/07/99. Of these initial pregnancies, there was a total of 14,676 foetuses, resulting in 14,062 live births and 13,988 children who were alive at 1 year of age.

When the oldest children were approximately 7 years of age, an attempt was made to bolster the initial sample with eligible cases who had failed to join the study originally. As a result, when considering variables collected from the age of seven onwards (and potentially abstracted from obstetric notes) there are data available for more than the 14,541 pregnancies mentioned above.

The number of new pregnancies not in the initial sample (known as Phase I enrolment) that are currently represented on the built files and reflecting enrolment status at the age of 18 is 706 (452 and 254 recruited during Phases II and III respectively), resulting in an additional 713 children being enrolled. The phases of enrolment are described in more detail in the cohort profile paper (see footnote 4 below).

The total sample size for analyses using any data collected after the age of seven is therefore 15,247 pregnancies, resulting in 15,458 foetuses. Of this total sample of 15,458 foetuses, 14,775 were live births and 14,701 were alive at 1 year of age.

A 10% sample of the ALSPAC cohort, known as the Children in Focus (CiF) group, attended clinics at the University of Bristol at various time intervals between 4 to 61 months of age. The CiF group were chosen at random from the last 6 months of ALSPAC births (1432 families attended at least one clinic). Excluded were those mothers who had moved out of the area or were lost to follow-up, and those partaking in another study of infant development in Avon.

*ALSPAC: Genotyping description*

ALSPAC children were genotyped using the Illumina HumanHap550 quad chip genotyping platforms by 23andme subcontracting the Wellcome Trust Sanger Institute, Cambridge, UK and the Laboratory Corporation of America, Burlington, NC, US. The resulting raw genome-wide data were subjected to standard quality control methods. Individuals were excluded on the basis of gender mismatches; minimal or excessive heterozygosity; disproportionate levels of individual missingness (>3%) and insufficient sample replication (IBD<0.8). Population stratification was assessed by multidimensional scaling analysis and compared with Hapmap II (release 22) European descent (CEU), Han Chinese, Japanese and Yoruba reference populations; all individuals with non-European ancestry were removed. SNPs with a minor allele frequency of < 1%, a call rate of < 95% or evidence for violations of Hardy-Weinberg equilibrium (P < 5x10^-7^) were removed. Cryptic relatedness was measured as proportion of identity by descent (IBD > 0.1). Related subjects that passed all other quality control thresholds were retained during subsequent phasing and imputation. 9,115 subjects and 500,527 SNPs passed these quality control filters.

*ALSPAC: Imputation description*

477,482 SNP genotypes in common between the sample of mothers and sample of children were combined. SNPs with genotype missingness above 1% due to poor quality were removed (11,396 SNPs removed). 321 subjects were removed due to potential ID mismatches. This resulted in a dataset of 17,842 subjects containing 6,305 duos and 465,740 SNPs (112 were removed during liftover and 234 were out of HWE after combination). Haplotypes were estimated using SHAPEIT (v2.r644) [[1](#_ENREF_1),[2](#_ENREF_2)] which utilises relatedness during phasing. A phased version of the 1000 genomes reference panel (Phase 1, Version 3) was obtained from the IMPUTE2 reference data repository (phased using SHAPEIT v2.r644, haplotype release date Dec 2013). Imputation of the target data was performed using IMPUTE V2.2.2 [[3](#_ENREF_3),[4](#_ENREF_4)] against the reference panel (all polymorphic SNPs excluding singletons), using all 2,186 reference haplotypes (including non-Europeans). This gave 17,842 mothers and children eligible for study with available genotype data. Subsequent consent withdrawals have left 17,825 individuals for study.

**References**

1. O'Connell J, Gurdasani D, Delaneau O, Pirastu N, Ulivi S, et al. (2014) A general approach for haplotype phasing across the full spectrum of relatedness. PLoS Genet 10: e1004234.

2. Delaneau O, Marchini J, Zagury JF (2011) A linear complexity phasing method for thousands of genomes. Nat Methods 9: 179-181.

3. Howie B, Marchini J, Stephens M (2011) Genotype imputation with thousands of genomes. G3 (Bethesda) 1: 457-470.

4. Howie BN, Donnelly P, Marchini J (2009) A flexible and accurate genotype imputation method for the next generation of genome-wide association studies. PLoS Genet 5: e1000529.
