# Supplementary figures and images for: Genetic architecture of human thinness compared to severe obesity
Source: PLoS Genet. 2019 Jan 24;15(1):e1007603. doi: 10.1371/journal.pgen.1007603 (PMC6345421; doi:10.1371/journal.pgen.1007603)

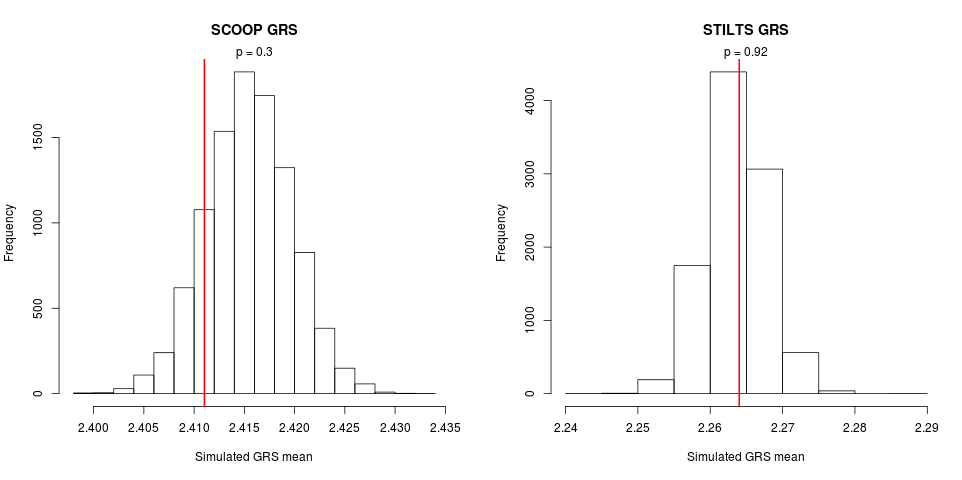

Supplement: S1 Fig — Histogram represents mean GRS scores for each BMI category across 10,000 simulations. Vertical red line highlights the observed value in real data. p = p-value of difference. (TIF) [file pgen.1007603.s003.tif]

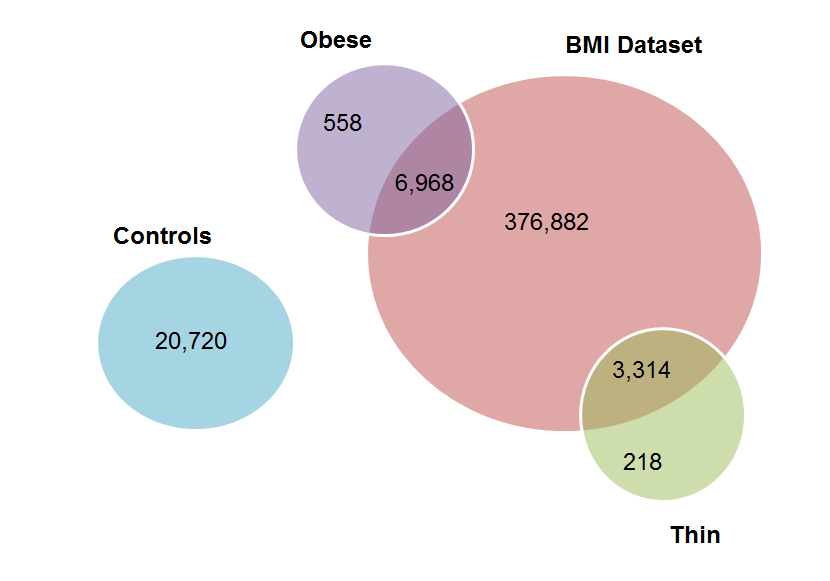

Supplement: S2 Fig — Venn Diagram showing sample numbers and overlap between UKBB sample sets used in genetic correlation (BMI dataset) and GWAS replication (obese, controls, thin) analyses. (TIF) [file pgen.1007603.s004.tif]

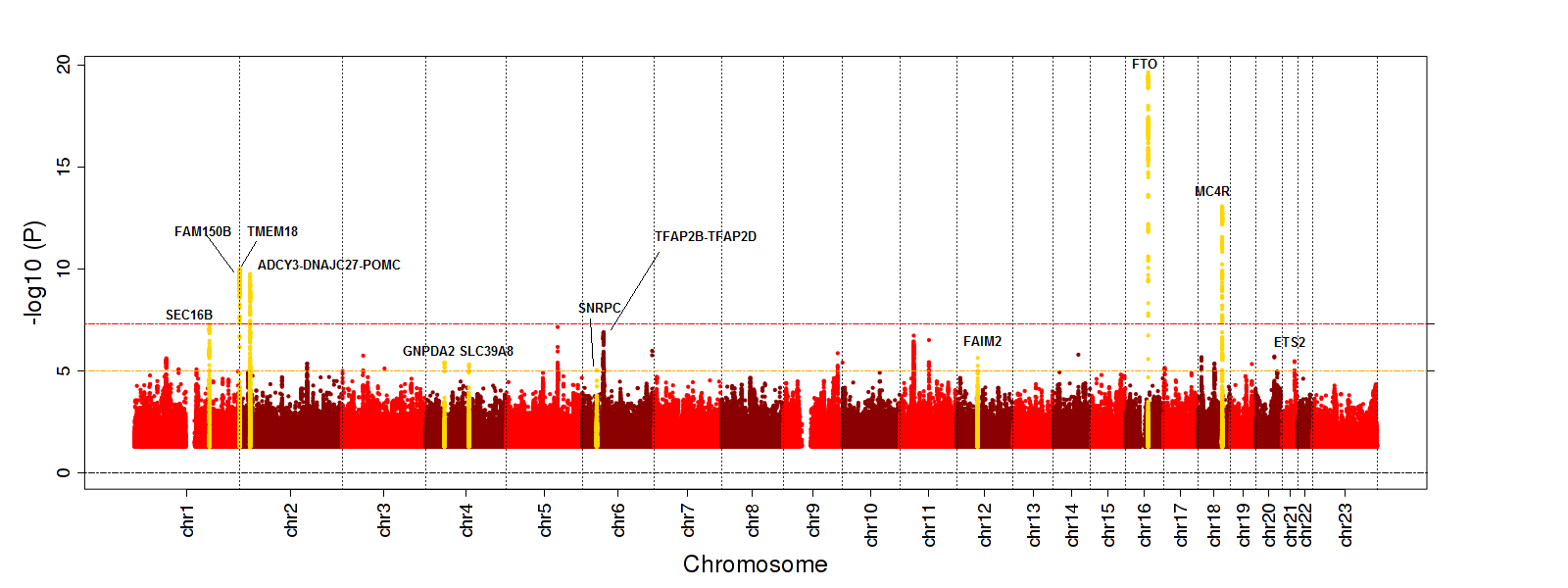

Supplement: S3 Fig — Manhattan plot produced in EasyStrata, red line indicates genome-wide significance threshold at p = 5x10^-08^. Orange line indicates discovery significance threshold at p = 1x10^-05^. Black labels highlight known BMI/obesity loci that were taken forward for replication and yellow peaks indicate those that met genome-wide significance after replication. (TIF) [file pgen.1007603.s005.tif]

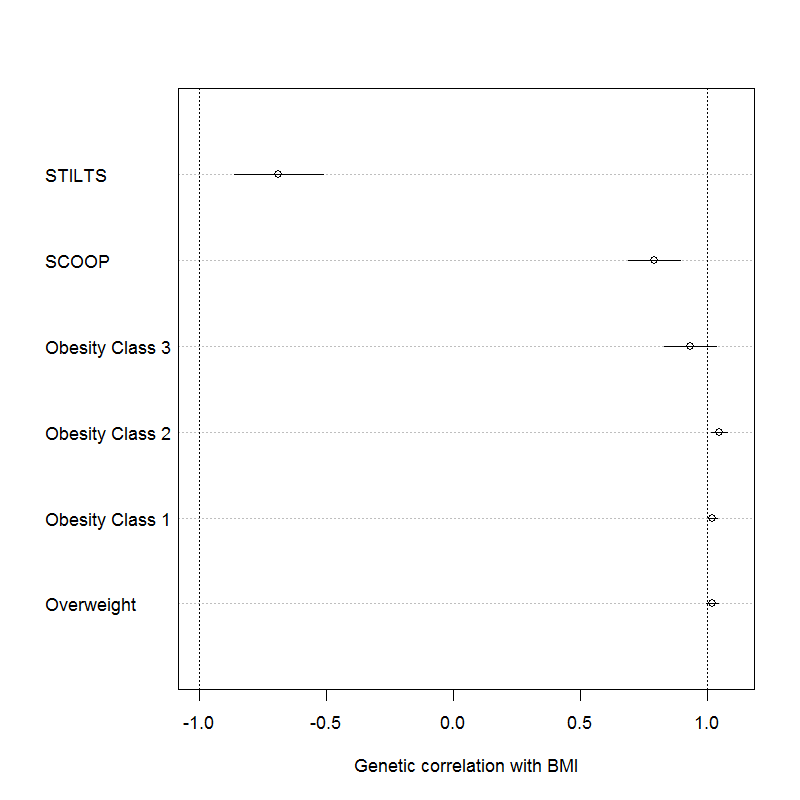

Supplement: S4 Fig — Genetic correlation estimates and 95% CI for severe early-onset childhood obesity (SCOOP), healthy persistent thinness (STILTS), Obesity Class 3, Obesity Class 2, Obesity Class 1 and Overweight. Dotted lines represent complete genetic correlation. (TIF) [file pgen.1007603.s006.tif]

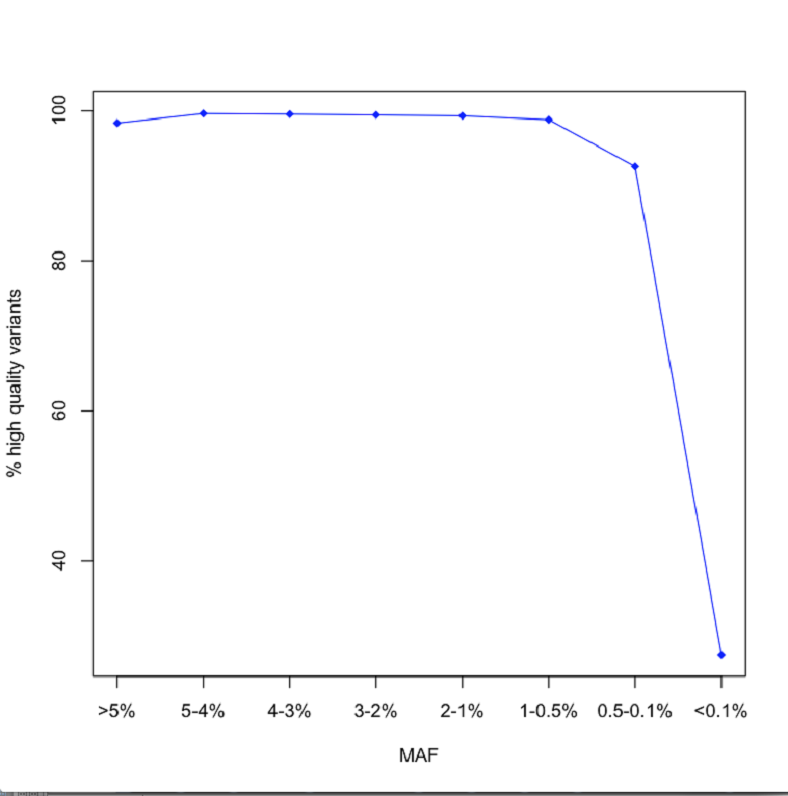

Supplement: S5 Fig — Percentage of variants with INFO score (r2)>0.4, as derived from the IMPUTE2 imputation algorithm, stratified by minor allele frequency across all samples (SCOOP, STILTS and UKHLS). (TIF) [file pgen.1007603.s007.tif]

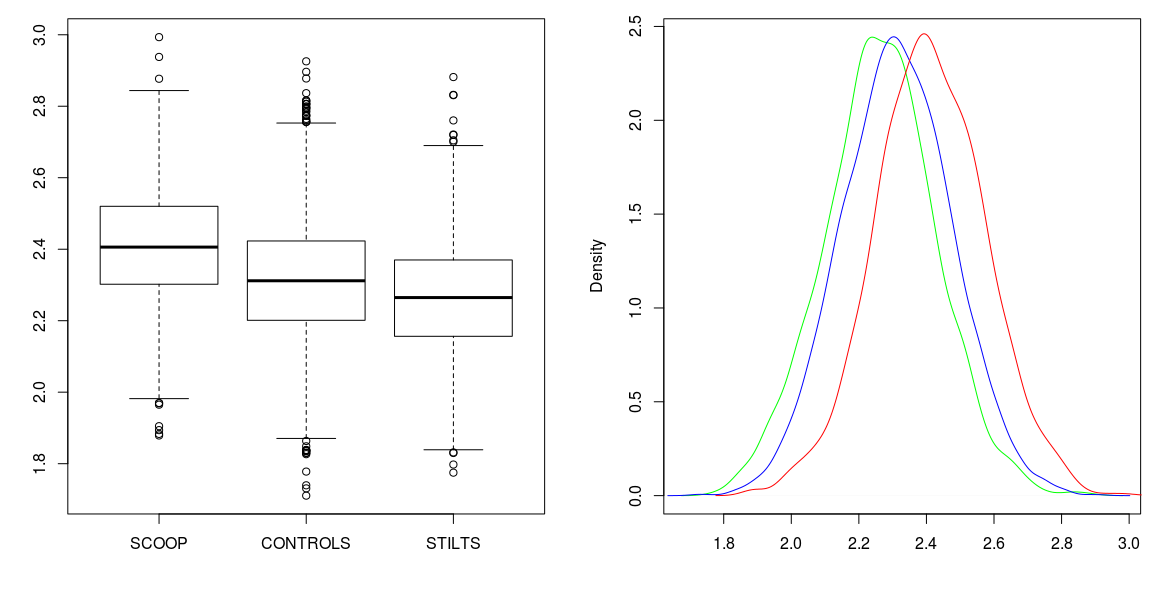

Supplement: S6 Fig — A weighted genetic risk score for each individual was obtained by summing genotype dosages multiplied by the effect (beta) estimates from GIANT for each of the 97 SNPs. To check the equal variance assumption, we used a box plot (left) and density plot (right). Density plot: Green = STILTS; Blue = UKHLS; Red = SCOOP. (TIF) [file pgen.1007603.s008.tif]

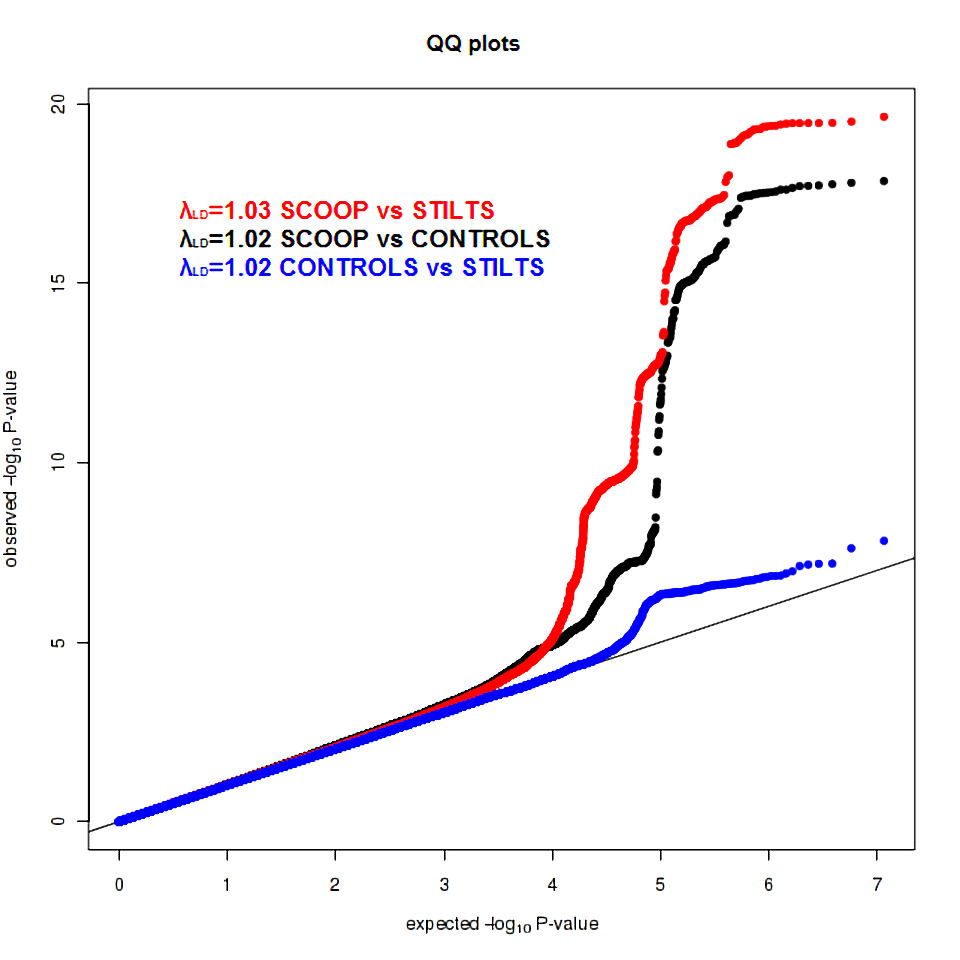

Supplement: S7 Fig — Q-Q plots of LD Score Regression-corrected p-values for the three analysis cohorts used for the discovery analysis, produced in EasyStrata. Red = SCOOP vs. STILTS; Black = SCOOP vs. UKHLS, Blue = STILTS vs. UKHLS. Variants passing QC and with MAF > = 0.5% are shown. LD Score regression intercept (λLD) values before correction are shown for each analysis. (TIF) [file pgen.1007603.s009.tif]

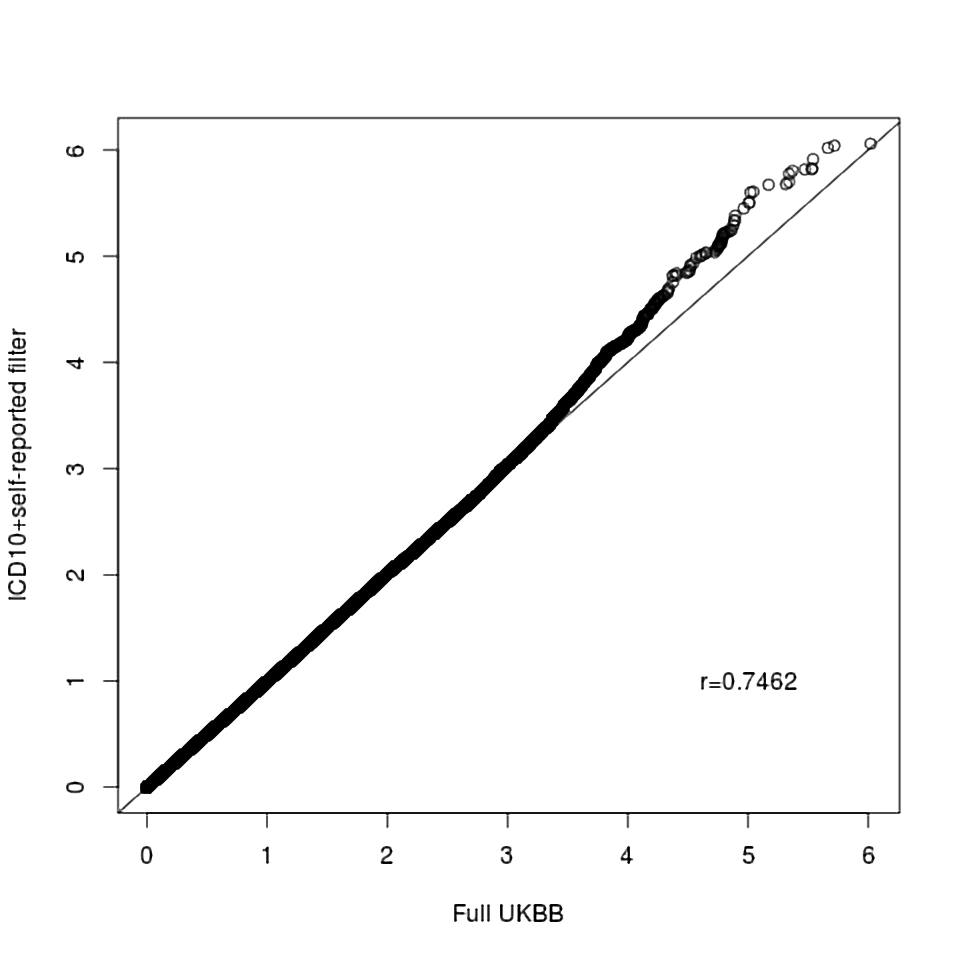

Supplement: S8 Fig — Q-Q plot using all thin individuals as cases (Full UKBB) and removing individuals based on ICD10 and self-reported data (ICD10+self-reported filter). Correlation for–log10 p-values is shown (r = 0.7462). (TIF) [file pgen.1007603.s010.tif]
